# Cytotoxic and anthelmintic potential of crude saponins isolated from *Achillea Wilhelmsii *C. Koch and *Teucrium Stocksianum *boiss

**DOI:** 10.1186/1472-6882-11-106

**Published:** 2011-11-03

**Authors:** Niaz Ali, Syed Wadood Ali Shah, Ismail Shah, Ghayour Ahmed, Mehreen Ghias, Imran Khan

**Affiliations:** 1 Department of Pharmacology, Institute of Basic Medical Sciences, Khyber Medical University, Peshawar, KPK, Pakistan; 2 Department of Pharmacy, University of Malakand, Chakdara, Dir, KPK, Pakistan; 3 Department of Biotechnology, University of Malakand, Chakdara, Dir, KPK, Pakistan

## Abstract

**Background:**

Saponins isolated from plant sources have a number of traditional and industrial applications. Saponins have pharmacological effects like anti-inflammatory, molluscicidal, antimicrobial, antispasmodic, antidiabetic, anticancer, anticonvulsant, anthelmintic, antitussive and cytotoxic activities. The current work describes the anthelmintic and cytotoxic activities of crude saponins of *Achillea Wilhelmsii *and *Teucrium Stocksianum *as these plants are rich with saponins.

**Methods:**

Brine shrimp cytotoxic activity of crude saponins was determined by Meyer et al. (1982) at test concentrations of 1000 μg/ml, 100 μg/ml, 10 μg/ml, 7.5 μg/ml, 5.0 μg/ml, 2.5 μg/ml and 1.25 μg/ml. Percentage mortality of test concentrations was determined. Similarly, in *vitro *anthelmintic activity was determined against roundworms, tapeworms and earthworms. Albendazole and piperazine citrate at concentration 10 mg/ml were used as standard anthelmintic drugs.

**Results:**

Crude saponins of *Achillea wilhelmsii *(CSA) and *Teucrium stocksianum *(CST) had, respectively, cytotoxic activity with LC_50 _values 2.3 ± 0.16 and 5.23 ± 0. 34 μg/ml. For in *vitro *anthelmintic activity, time for paralysis and death of parasites (parasiticidal activity) was noted. At concentration 40 mg/ml, crude saponins of *Achillea wilhelmsii *are 1.96 and 2.12 times more potent than albendazole against *Pheretima posthuma *and *Raillietina spiralis*, respectively. Similarly, at concentration 40 mg/ml, crude saponins of *Teucrium stocksianum *(CST) has 1.89, 1.96 and 1.37 times more parasiticidal activity than albendazole against *Pheretima posthuma, Raillietina spiralis *and *Ascardia galli*, respectively.

**Conclusion:**

Crude saponins of *Achillea wilhelmsii *and *Teucrium stocksianum *have cytotoxic and anthelmintic activity. The crude saponins may be excellent sources of cytotoxic and anthelmintic constituents that warrant its isolation and purification for new drug development.

## Background

Saponins are a large family of structurally related heterosides compounds of steroid or triterpenoid aglycone (sapogenin) linked to one or more oligosaccharide moieties by glycosidic linkages. The carbohydrate moiety consists of pentose(s), hexose(s) or uronic acid(s) [1,2]. Saponins are classified, according to their aglycone skeleton, as non-steroidal saponins, steroidal saponins and steroidal amines that are also referred to as steroidal alkaloids [3].

Saponins have a number of traditional and industrial applications [4-6]. Saponins from plants sources are also responsible for some pharmacological effects like anti-inflammatory [7], molluscicidal [8], antimicrobial [9], antispasmodic [10], antidiabetic and anticancer [11], hypocholesterolemic [12], antioxidant [13], anticonvulsant and analgesic [14], anthelmintic, antitussive and cytotoxic activities [15].

*Achillea wilhelmsii *(Local name: Zawal) belongs to Asteraceae, which contains alkaloids, flavonoids, terpenoids, volatile oils, sesquiterpenelactones and saponins [16]. While *Teucrium stocksianum *belongs to family Lamiaceae, which contains phytochemicals like carbohydrates, proteins and amino acids, tannins, flavonoids, sterols and saponins. It gave negative tests for alkaloids, anthraquinone glycosides and cardiac glycosides [17]. Based on the reported literature for different saponins as cytotoxic and anthelmintic agents, the current work is carried out to screen the crude saponins of *Achillea wilhelmsii *and *Teucrium stocksianum *for possible cytotoxic and anthelmintic activity.

## Methods

### Plant Materials

Plant *Achillea wilhelmsii *was purchased from the local market of Nasir Bagh, Board district Peshawar, KPK. *Teucrium stocksianum *was collected from nearby hills of University of Malakand in the month June - July 2009. The plants were authenticated by Professor Dr. Jehandar Shah, vice chancellor Shaheed Benazir Bhuto University, Sheringal Dir Upper, KPK. Voucher specimens, respectively, AW-2009 for *Achillea wilhelmsii *and T-01-2009 for *Teucrium stocksianum *were deposited in the herbarium of Department of Botany, University of Malakand. Ethical Committee of the department of pharmacy approved the experimental protocols as per animal byelaws 2008 of the University of Malakand "Scientific Procedures Issue I".

### Preparation of extract and the crude saponins

170 grams of powdered materials of aerial parts of *Achillea wilhelmsii *and *Teucrium stocksianum *were extracted with petroleum ether by successive extraction in Soxhlet apparatus followed by extraction with commercial grade methanol. The solvents were subjected to rotary evaporation under vacuum to obtain dry semi solid extracts. The methanol extracts of both plants were further fractionated with *n*-butanol and water, in equal proportion, to get the *n*-butanol fraction. The crude saponins were precipitated with ether yielding 12.35 g of crude saponins of *Achillea wilhelmsii *(CSA) and 9.80 g of crude saponins of *Teucrium stocksianum *(CST) [18].

### Drugs and chemicals

All the reagents used were of analytical grade (E. Merck). Piperazine citrate (GSK) and albendazole (GSK) were used as standard reference drugs in the experiments at concentration 10 mg/ml.

### Statistical analysis and calculations

Statistical analysis was performed at 95% confidence interval. P value equal to or less than 0.05 was considered as significant. Microsoft XL sheet and Graph Pad prism were used to calculate mean, SEM and draw the curves for EC_50 _and LC_50_.

#### Brine Shrimp Cytotoxicity

Brine shrimp cytotoxic activity of crude saponins was determined as described by Meyer et al. (1982) with some modifications. Briefly describing, brine shrimp eggs (*Artemia salina*) were placed on one side of a small tank which was filled with sea water, covered with aluminum foil, and fully aerated. After 48 h incubation at room temperature and under illumination, the resulting nauplii (larvae) were attracted to the other side of the tank with a light source. The nauplii were collected with the help of a dropper.

Stock solutions (10 mg/ml) of CSA and CST were prepared by dissolving 20 mg of each sample in 2 ml of methanol. From the stock solution, 1000 μg/ml, 100 μg/ml, 10 μg/ml, **7**.5 μg/ml, 5.0 μg/ml, 2.5 μg/ml and 1.25 μg/ml were prepared by taking 500 μl, 50 μl, 5 μl, 3.75 μl, 2.5 μl, 1.25 μl and 0.625 μl, respectively. The solvents were evaporated from the vials by exposing to evaporation for 24 hours. 2 ml of sea water was added and ten brine shrimps nauplii were transferred with the help of a dropper to each sample vial and the volume of sea water was adjusted to 5 ml. Negative control experiments contained 5 ml of sea water and ten brine shrimps. Survivors were counted after 24 h [19]. Percentage mortality of test concentrations and control was determined using the equation: % mortality = (no. of dead nauplii/initial no. of live nauplii) × 100. Experiments for each concentration were performed in triplicate.

LC_50 _values less than 100 ppm (100 μg/mL) were considered significant.

#### Anthelmintic activity

Adult roundworms (*Ascaridia galli*), tapeworms (*Raillietina spiralis*) and earthworms (*Pheretima posthuma *L. Vaill) were used to evaluate in *vitro *anthelmintic activity. The earth worms were collected from swampy water near the new boys' hostel, University of Malakand, Dir, KPK, Pakistan. Roundworms and tapeworms were obtained from the intestines of freshly slaughtered fowls. Their intestines were treated with normal saline solution to remove all the fecal matters. The worms were collected after dissection of intestines and maintained in normal saline solution, having an average size of roundworms, tapeworms and earthworms as 5-7 cm, 6-7 cm and 7-8 cm, respectively. The assay was performed by using adult earthworms in *vitro *because the earthworm has high resemblance, both anatomically and physiologically, with the intestinal roundworm parasite *Ascaris lumbricoides *of human beings. The prelabeled extracted saponins from both plants were prepared in distilled water at concentrations of 10, 20 and 40 mg/ml. Six worms, approximately of equal size, each of *Pheretima posthuma, Raillietina spiralis *and *Ascaridia galli*, were placed in petri dishes. Each petri dish contained 25 ml of test solution of the extracts. For reference standards, Albendazole and Piperazine citrate (10 mg/ml each) were used as positive controls, and distilled water was used as the negative control.

The experiments were run in triplicate. Before starting the experiments, standard drugs and test solutions were freshly prepared. Time for paralysis was recorded when no movement was observed except when shaken vigorously, whereas time of death was recorded when the worms did not show any movement by vigorous shaking nor when dipped in warm water (50°C) [20].

## Results and discussion

**Figure 1:**
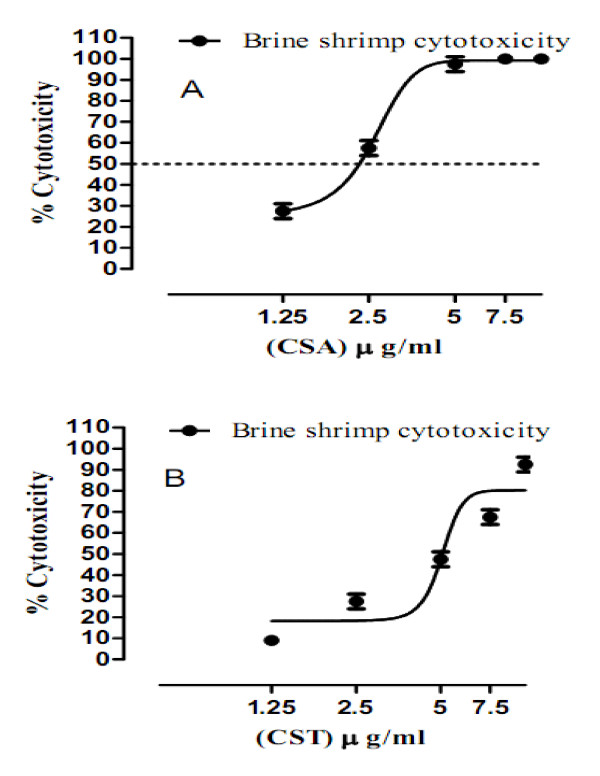
**Cytotoxic activity of crude saponins of *Achillea wilhemsii* and *Teucrium stocksianum***. (A): Cytotoxic activity of crude saponins of *Achillea wilhelmsii *(CSA). (B): Cytotoxic activity of crude saponins of *Teucrium stocksianum *(CST).

Crude saponins of *Achillea wilhelmsii *(CSA) were very potent and killed all the shrimps at concentrations 1000, 100, 10 and 7.7 μg/ml (Table 1). Plotting concentration response curves for 5, 2.5 and 1.25 μg/ml, LC_50 _value is 2.30 ± 0.16 μg/ml (n = 3) (Figure 1A). 100% cytotoxic activity was shown by crude saponins of *Teucrium stocksianum *at test concentrations 1000 and 100 μg/ml (Table 1). Similarly, plotting concentration response curves for CST at concentrations 10, 7.5, 5.0 2.5 and 1.35 μg/ml, LC_50 _was 5.23 ± 0.34 μg/ml (Figure 1B). While comparing the LC_50 _values, it is evident that crude saponins of *Achillea wilhelmsii *were more potent (more than 2 times) than crude saponins of *Teucrium stocksianum*. Hence, it is deduced that the crude saponins of both species have cytotoxic constituents. Based on the previous literature reports for positive correlation between the brine shrimp cytotoxicity assay and human KB cell line (human nasopharyngeal carcinoma), the current work warrants for isolation of these anticancer constituents present in the plants [21].

**Table 1:** Brine shrimp cytotoxicity of crude saponins of *Achillea wilhelmsii *and *Teucrium stocksianum*.

Sample	**Conc**. (μg/ml)	No of brine shrimps taken	No of brine shrimps killed	LC_50 _(μg/ml)
	1000	10	10 ± 0	
	100	10	10 ± 0	
	10	10	10 ± 0	
**CSA**	7.5	10	10 ± 0	2.3 ± 0.16
	5	10	9.67 ± 0.58	
	2.5	10	06 ± 1	
	1.25	10	03 ± 1	

	1000	10	10 ± 0	
	100	10	10 ± 0	
	10	10	9.8 ± 0.76	
**CST**	7.5	10	7 ± 0.5	5.23 ± 0.34
	5	10	5 ± 0.5	
	2.5	10	2.9 ± 0.36	
	1.25	10	1 ± 0.14	

Key:

CSA = Crude Saponins of *Achillea wilhelmsii*.

CST = Crude Saponins of *Teucrium stocksianum*.

**Figure 2:**
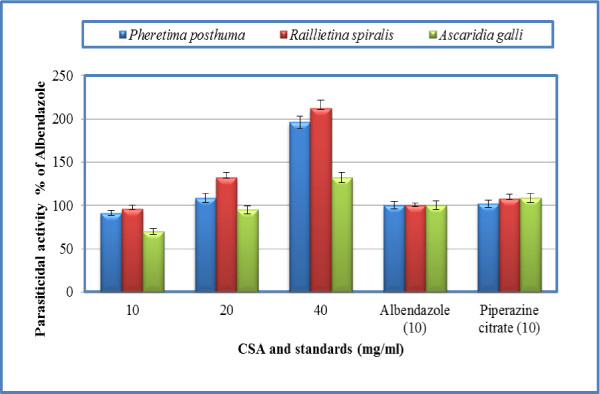
**Parasiticidal activity of crude saponins of *Achillea wilhelmsii *(CSA) as % of albendazole**.

**Figure 3:**
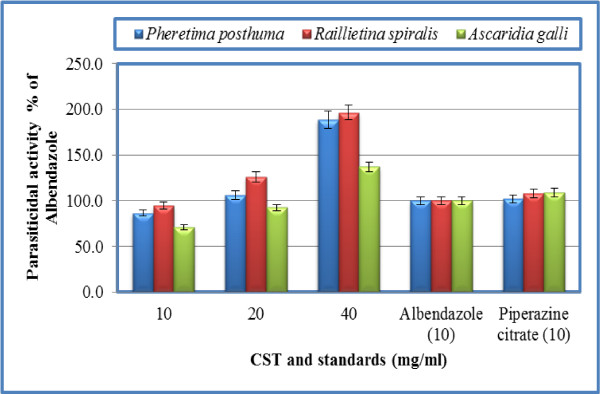
**Parasiticidal activity of crude saponins of *Teucrium stocksianum *(CST) as % of albendazole**.

Results for anthelmintic activity against the test parasites are mentioned in Table 2. The crude saponins of *Achillea wilhelmsii *showed dose dependent response against the test parasites. Time for paralysis and death (parasiticidal activity) are shown in Table 2. The crude saponins of *Achillea wilhelmsii *showed comparable efficacy to piperazine citrate against *Pheretima posthuma *at concentration 20 mg/ml. Quantifying the effects as % of albendazole, the results are expressed in Figure 2. At concentration 40 mg/ml of crude saponins of *Achillea wilhelmsii*, the parasiticidal activity is 1.96 and 2.12 times more potent than albendazole against *Pheretima posthuma *and *Raillietina spiralis*, respectively. Against *Ascaridia galli*, the parasiticidal activity was comparable with albendazole at concentrations 10 and 20 mg/ml; however, at concentration 40 mg/ml, activity was 1.32 times that of albendazole (Figure 2). Similarly, at concentration 40 mg/ml, crude saponins of *Teucrium stocksianum *have 1.89, 1.96 and 1.37 times parasiticidal activity of albendazole against *Pheretima posthuma, Raillietina spiralis *and *Ascaridia galli*, respectively (Figure 3). From the above results, it is clear that the crude saponins of *Achillea wilhelmsii *and *Teucrium stocksianum *have excellent anthelmintic activity. Other interesting findings of the study are that albendazole and piperazine citrate have comparable in *vitro *efficacy against the test parasites.

**Table 2:** Results of anthelmintic activity of crude saponins of *Achillea wilhelmsii *and *Teucrium stocksianum*.

	Time taken for paralysis (P) and death (D) in minutes						
**Sample/Groups**	**Conc**. **mg/ml**	***Pheretima posthuma*** **(Earthworm)**		***Raillietina spiralis*** **(Tapeworm)**		***Ascaridia galli*** **(Roundworm)**	

		**(P)**	**(D)**	**(P)**	**(D)**	**(P)**	**(D)**
	
	10	24	56	28	55	21	53
**CSA**	20	17	47	17	40	16	39
	40	09	26	11	25	11	28

	10	27	59	28	56	24	52
**CST**	20	20	48	19	42	18	40
	40	11	27	14	27	12	27

Albendazole	10	19	51	19	53	12	37

Piperazine citrate	10	17	50	16	49	10	34

Negative control	--	--	--	--	--	--	--

Key:

CSA = Crude Saponins of *Achillea wilhelmsii*.

CST = Crude Saponins of *Teucrium stocksianum*.

## Conclusion

Crude saponins of *Achillea wilhelmsii *and *Teucrium stocksianum *have cytotoxic and anthelmintic activity. The crude saponins may be excellent sources of cytotoxic and anthelmintic constituents that warrant their isolation and purification from the medicinal plants for a more thorough investigation.

## Competing interests

The authors declare that they have no competing interests.

## Authors' contributions

NA participated in writing manuscript and data interpretation. The research scholars were also guided for laboratory work. SWA participated in brine shrimps study and data acquisition. IS participated in data acquisition. GA participated in data acquisition. MG helped in literature survey and data acquisition of anthelmintic activity. IK helped in data acquisition of anthelmintic activity. All authors read and approved the final manuscript.

## Pre-publication history

The pre-publication history for this paper can be accessed here:

http://www.biomedcentral.com/1472-6882/11/106/prepub
